# Efficacy of a low dose fipronil bait against blacklegged tick (*Ixodes scapularis*) larvae feeding on white-footed mice (*Peromyscus leucopus*) under laboratory conditions

**DOI:** 10.1186/s13071-020-04258-0

**Published:** 2020-07-31

**Authors:** David M. Poché, Gregory Franckowiak, Tyler Clarke, Batchimeg Tseveenjav, Larisa Polyakova, Richard M. Poché

**Affiliations:** Genesis Laboratories, Inc., Wellington, CO USA

**Keywords:** *Borrelia burgdorferi* (*sensu stricto*), Blacklegged ticks, *Ixodes scapularis*, White-footed mice, *Peromyscus leucopus*, Fipronil bait, Acaricides, Systemic insecticides, Vector control

## Abstract

**Background:**

Lyme disease is the most prevalent vector-borne disease in the USA with cases continuing to increase. Current control measures have not been shown to be impactful, and therefore alternatives are needed. Treating pathogen reservoirs with low dose systemic acaricides in endemic areas may provide a useful tool for disrupting the cycle of the vector and pathogen. The purpose of this study was to determine the efficacy of a 0.005% fipronil bait, presented orally to white-footed mice, in controlling blacklegged tick larvae (larvae).

**Methods:**

Sixty mice were assigned to 3 treatment groups and three untreated control groups. All individually housed mice in treatment groups were exposed to 0.005% fipronil bait for 48 hours. Larvae were manually applied to mice within feeding capsules at one of three timepoints: Day 1, Day 9 and Day 15 post-exposure. For 4-days post-tick attachment, replete larvae were collected from water moats underneath each cage and attached larvae were observed by microscopy. Plasma from 4 treated mice at Day-1, Day 13 and Day 19, and 4 control mice (*n *= 16) was collected to obtain fipronil plasma concentrations (CP).

**Results:**

Fipronil bait did not appear to produce neophobia in mice, as the amount of bait eaten at 24- and 48-hours exposure did not differ significantly. The 48-hour fipronil bait exposure prevented 100% of larvae from feeding to repletion at Day 1, Day 9 and Day 15 post-treatment. Within the treatment groups, all larvae observable within the capsules expired and were prevented from detaching by Day 4. In contrast, within the control groups a total of 502 replete larvae were collected from moats and 348 larvae observable within the capsules successfully detached. CP averaged 948.9, 101.2 and 79.4 ng/ml for mice euthanized at Day 1, Day 9 and Day 15, respectively. No fipronil was detected in control mice.

**Conclusions:**

We provide early indication that low dose fipronil bait, orally presented to white-footed mice, can effectively control blacklegged tick larvae. Future research should modify the exposure duration and post-exposure tick attachment timepoints to simulate various field scenarios under which successful efficacy might be obtained. Low dose fipronil bait could provide a cost-effective, practical means of controlling blacklegged ticks and other arthropod vectors.
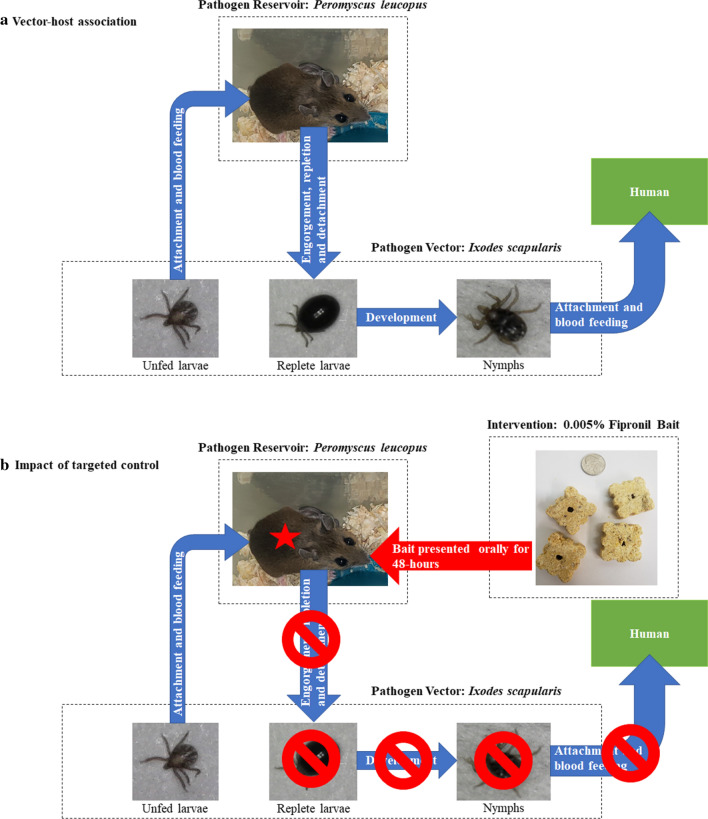

## Background

Lyme disease is the most prevalent vector-borne disease transmissible to man in the USA and cases have continued to increase from 2001 to present [1, 2], with over 300,000 cases estimated to occur annually and a geographical distribution that is continually expanding [2, 3]. In the USA, human instances of Lyme disease are most commonly reported in the midwestern/north-central and northeastern regions [1, 4] where the blacklegged tick (*Ixodes scapularis*) serves as the primary pathogen vector for the Lyme disease spirochete (*Borrelia burgdorferi* (*sensu stricto*)) with the white footed mouse (*Peromsyscus leucopus*) serving as a primary reservoir host for immature blacklegged ticks in these regions [5].

Initial Lyme disease symptoms are flu-like and accompanied by a specific rash present in 60–80% of patients referred to as erythema migrans [6]. If cases are not quickly treated with antibiotics, infection can spread to joints, the heart, and nervous system [4]. Lyme disease is difficult to diagnose, partly because of clinical non-specificity [7], and it is not uncommon for patients treated with 2–4 weeks of antibiotics to suffer from post-treatment Lyme disease syndrome (PTLDS) which can last for more than six months and may last for many years [8]. The economic burden of Lyme disease is substantial with the cost to the American Healthcare System having been estimated to be between $712 million and $1.3 billion per year, and annual fees per patient suffering from Post-Treatment Lyme Disease Syndrome (PTLDS) are estimated to be $3000 and $3800 [9]. Thus, the disease is still of understandable importance in the USA and alternative methods of disease prevention and vector control should continue to be investigated to alleviate the significant burden placed upon medical practitioners and patients.

Acaricide application methods aimed at controlling blacklegged ticks are among the more promising approaches for Lyme disease prevention. However, large-scale pesticide application has been hindered largely as a biproduct of environmental concerns [10, 11]. Traditional methods such as dusting or blanket spraying require a large volume of acaricide and a high concentration of active ingredient in the formulations [11] that are exponentially higher than what would be required to control tick larvae and nymphs [12–14]. The greatest risk factors for Lyme disease are exposure to infected *Ixodes* spp. ticks and reservoir host animals, particularly the white-footed mouse, in woody, grassy areas [15].

The life-cycle of blacklegged ticks takes approximately two years to complete and is composed of four life stages (eggs, larvae, nymphs and adults). After the eggs hatch, they require a blood meal at each subsequent life stage to survive and develop [16]. Larvae hatch pathogen-free in the summer and take a blood meal from small rodents, host-feeding heavily on white-footed mice. It is at this point that they acquire the *B. burgdorferi* (*s.s.*) spirochete. Once larvae have blood-fed to repletion, they detach from the host and begin molting. Infected larvae will typically emerge to feed as nymphs the following spring. The risk of human exposure to Lyme disease is shown to be a function of the local abundance of nymphal and adult ticks [17] and is strongly correlated with the density of spirochete-infected ticks in the areas surrounding residences [18]. Nymphs are believed to be the primary pathogen reservoir responsible for *B. burdorferi* transmission [2] and may be responsible for as much as 90% of Lyme disease cases each year [19]. Risk of *B. burgdorferi* (*s.s.*) infection is shown to increase in response to increases in infected nymphs [20]. Given the issues regarding traditional acaricide application and *B. burgdorferi* (*s.s.*) risk factors such as density of nymphs and proximity of host animals, strategies such as targeting white-footed mice with an acaricide, aimed at reducing larval tick density, could prove beneficial. A targeted approach would markedly reduce the amount of active ingredient being applied in the field, which would pose a reduced risk to non-target organisms. Significantly reducing larvae feeding on white-footed mice could reduce the density of incoming nymphs and reduce *B. burgdorferi* (*s.s.*) infection rates.

Fipronil is a phenylpyrozol that interferes with arthropod central nervous systems by blocking the GABA-gated and glutamate-gated chloride channels [21]. It has shown promise in controlling several arthropod pests and has been explored for use in tick control. One such method being explored is the use of the Select TCS bait box (EPA Est. No. 85306-CT-001). This approach involves a bait station, which is filled with attractive bait and is fitted with a cotton or felt wick treated with a concentration of 0.70–0.75% fipronil that is topically applied to white-footed mice entering the bait station [22–24]. While the approach has reduced ticks under experimental conditions, its deficiencies have hindered widespread use. The bait station is noted as being costly and needing to be periodically replaced [24]. Mice are also able to avoid the wick or will routinely remove them for use as bedding material [23]. Thus, many mice entering the bait stations to feed are not inoculated and use of a single wick with fipronil requires bait stations be replaced. Additionally, the product is a restricted use pesticide, meaning that only licensed pest control professionals may use it, which further limits utilization. Bait stations/boxes will need to be baited regardless of the route of acaricide administration (i.e. topical, oral). Thus, a practical and cost-effective solution would be to load the bait stations with a low dose acaricide bait for oral uptake by white-footed mice.

An oral acaricide bait would act systemically, meaning the acaricide would be absorbed by larvae during blood-feeding. Under field conditions, this approach could disrupt the *B. burgdorferi* (*s.s.*) cycle by preventing blacklegged tick larvae from feeding to repletion and detaching, subsequently reducing the number of nymphs that humans would typically be exposed to. During previous laboratory research, fipronil baits, at concentrations of 0.097% and 0.0485%, presented orally to house mice (*Mus musculus*) were 100% efficacious in preventing larval blacklegged ticks from feeding to repletion when larvae were fed on mice immediately following a 48-hour exposure period [25]. A more recent laboratory study evaluated the use of 0.005% and 0.0015% fluralaner baits presented orally to deer mice (*Peromyscus maniculatus*) [26]. When mice were exposed to these baits for 24 hours, at Day-2 post-exposure, blacklegged tick larvae were reduced by up to 97% and 94%, respectively.

The results of these above studies are useful; however, additional research is warranted. The above studies utilized house mice [25] and deer mice [26], respectively. While these studies provide insights regarding the potential for blacklegged tick control, additional mammalian species should be investigated, particularly the white-footed mouse. Additional mammalian reservoirs such as chipmunks and shrews may be significant hosts for immature ticks and may be pathogen reservoirs in certain locations, a belief speculated to have contributed to the failure of some white-footed mouse treatment schemes [27]. However, given the fact that the white-footed mouse is a well-established primary pathogen reservoir in the midwestern and northeastern USA, and the fact that fipronil-based treatments targeting white-footed mice have shown promise in controlling ticks in both laboratory and field settings, we would argue that it is currently the most appropriate rodent model for assessing the potential for a low dose fipronil bait to control immature blacklegged ticks in the northeastern and midwestern USA.

Additionally, the ticks attached to mice during these studies were allowed to free-feed on mice, making direct tick observation difficult. Oral acaricides act systemically, causing ticks to expire during blood-feeding and many remain attached to the host. Feeding capsules have become a standard in tick colonies to facilitate blood-feeding and promote development and oviposition [28–31] and could provide a useful method for observing and confirming the mortality of attaching ticks. The use of a capsule may also prevent ticks from being removed and damaged during grooming, an issue previously reported by other researchers [26, 32].

The above oral acaricide baits were efficacious in controlling blacklegged tick larvae. However, palatability was noted to be an issue with the fipronil baits [25]. The concentrations in these baits were 0.097% and 0.0485%, which were much higher than necessary to control ticks and may have contributed to taste aversion. More recent field experiments involving fleas suggest that the fipronil concentration in these baits could be reduced markedly. In Northern Colorado, application of a 0.005% fipronil bait resulted in > 95% control of *Oropsylla* spp. fleas infesting black-tailed prairie dogs up to 52 days post-fipronil bait application [33]. During another field trial in southeastern Kazakhstan, 0.005% fipronil bait application resulted in 100% removal of *Xenopsylla* spp. fleas infesting great gerbils (*Rhombomys opimus*) up to 80-days post-fipronil bait application [34]. Additional laboratory bioassays have indicated 0.005% fipronil to effectively control adult and larval phlebotomine sand flies (*Phlebotomus argentipes)* [35]. The effectiveness of low dose fipronil baits against fleas and phlebotomine sand flies infesting rodents suggest that the potential to control blacklegged ticks should be evaluated. The nominal concentration in 0.005% fipronil baits is roughly 19.4× and 9.7× lower than that of the fipronil baits previously utilized in tick control [25], which would pose reduced risk to white-footed mice and non-target organisms. If properly evaluated, a 0.005% oral fipronil bait could prove to be a useful addition to integrated tick management programs.

The study conducted by Pelletier et al. [26] estimated efficacy two days after tick attachment, only. Larvae feed for ~4 days after which replete larvae will drop off and begin molting [36]. Therefore, ticks not succumbing to acaricides at Day-2 post-tick attachment may still expire prior to feeding to repletion and detaching. It would be useful to evaluate the efficacy of acaricides over a duration spanning the ~4-day larval tick attachment period.

**Fig. 1:**
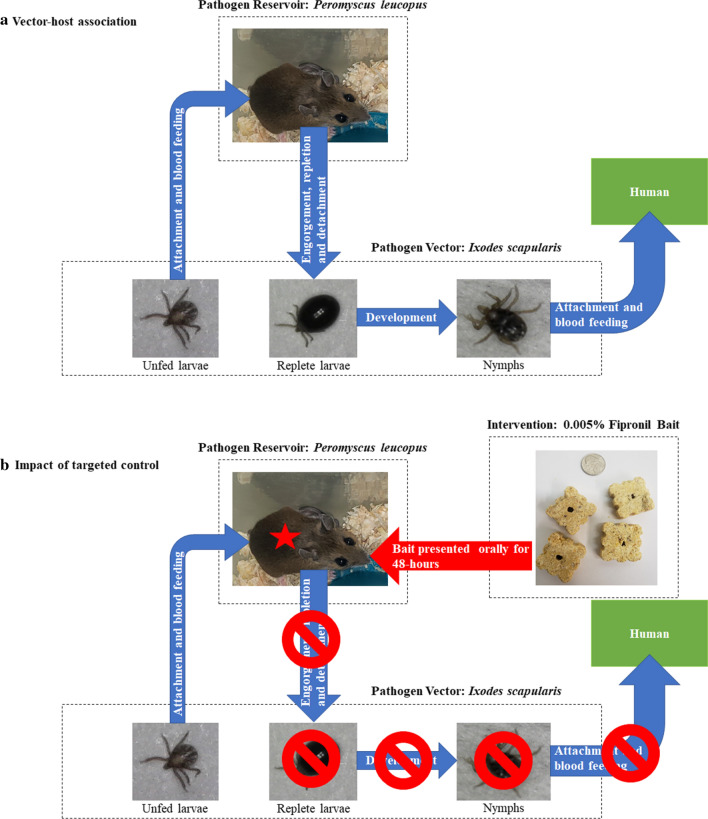
**a** Vector-host association: Larvae attach to the white-footed mouse and begin blood-feeding for ~4 days. Fully engorged, replete larvae drop from the host and begin nymph development. Infected nymphs can then bite humans. **b** The impact of fipronil bait consumption by white-footed mice on development of blood-feeding larvae: Larvae blood-feeding on mice that consume fipronil bait expire and are prevented from feeding to repletion and detaching, subsequently preventing nymph development and reducing the risk of human nymph bites

The purpose of this laboratory study was to determine the efficacy of an oral acaricide, paraffin rodent bait containing 0.005% fipronil, presented to white-footed mice for 48 hours, in controlling larval blacklegged ticks allowed to attach to mice at Day 1, Day 9 and Day 15 post-bait exposure. We hypothesized that fipronil bait would successfully control blacklegged tick larvae by preventing them from feeding to repletion and detaching, and subsequently preventing molting and nymph development (Fig. 1). We estimated the effectiveness of the bait by collecting blacklegged tick larvae which fed to repletion and detached up to 4 days post-tick attachment and by observing larvae attached to the host *via* microscopy. Our target efficacy was a minimum of 90% success in preventing larvae from feeding to repletion and detaching, relative to control groups, following the guidelines of the United States Environmental Protection Agency (EPA) who require a minimum efficacy of at least 90% against ectoparasites [37].

## Methods

This research was performed under laboratory conditions at Genesis Laboratories, Inc. (Genesis) in Wellington, Colorado.

### White-footed mice

All procedures performed during this study involving white-footed mice, and the test protocol, were approved by the Genesis Institutional Animal Care and Use Committee (IACUC) (March 18, 2019) and followed AWA and Genesis IACUC policies (Study No. 19001).

Test mice were from an outbred white-footed mouse breeding colony originally initiated using 20 mice received from the *Peromyscus* Genetic Stock Center (University of South Carolina). Mice were housed separately in order to accurately estimate individual consumption and to reduce the probability of mice removing attached ticks. Mice were housed in screen-bottom metal cages with a surface area of ~550 cm^2^ in accordance with EPA recommendations [38].

### Blacklegged ticks

Blacklegged tick larvae (larvae) feed heavily on white-footed mice, which are a key pathogen reservoir. Larvae were acquired from the Oklahoma State Tick Rearing Facility (Stillwater, OK, USA) and were maintained in a regulated insectary. Temperatures in the insectary were maintained at ~20–24 °C with a photoperiod of 12 hours light:12 hours dark. Larvae were housed in a rearing desiccator, with temperature and relative humidity maintained at ~21–22 °C and > 90–99%, respectively. A saturated solution of potassium sulfate (140 g/l water) was added to the desiccator to maintain humidity and prevent mold growth. Humidity and temperature were monitored daily.

### Fipronil bait

The fipronil bait was manufactured as a solid bait block containing a nominal concentration of 0.005% fipronil (50 mg/kg) (Scimetrics Limited Corp., Wellington, Colorado). The bait formulation consisted largely of paraffin wax and was of limited nutritional value. Before presenting fipronil bait to mice, the nominal fipronil concentration (0.005%; 50 mg/kg) was confirmed by separating matrix interferences using a validated method of high-performance liquid chromatography (HPLC) and ultraviolet (UV) detection. The mean fipronil concentration was 47.8 ± 4.65 mg/kg (CV: 9.73%; Recovery: 95.6%). The bait was within the requirements outlined by the EPA for test substance concentration (±10%).

### Experimental design

#### Pre-exposure (Acclimation)

Sixty mice were individually housed and acclimated to test conditions for 7 days prior to fipronil bait exposure. During acclimation, mice were observed daily for general health and were provided fresh water and commercial laboratory rodent diet *ad libitum*. A veterinarian inspected the mice prior to fipronil bait exposure to ensure that all were suitable for the study.

#### Group assignment

The sex, weight, and parental lineage of mice were indicators used to randomly assign them to test groups using a sequence generator (random.org). Each group was assigned 5 males and 5 females. Test group mice were distinguished by (i) exposure to fipronil bait (Yes/No); and (ii) the timepoint post-exposure during which larvae were allowed to attach and feed (Table 1).

**Table 1:** The six test groups utilized and the attachment timepoints

Test group ID*	No. of mice with larvae attached		
	Timepoint post-exposure		
	Day 1	Day 9	Day 15
Treatment Day 1	10	–	–
Treatment Day 9	–	10	–
Treatment Day 15	–	–	10
Control Day 1	10	–	–
Control Day 9	–	10	–
Control Day 15	–	–	10

#### Exposure

At the end of acclimation, commercial rodent diet was removed from the cages of each mouse within TDay1, TDay9 and TDay15 and replaced with ~20 g fipronil bait. Mice were provided the fipronil bait exclusively for 48-h. A multi-day exposure period was selected to address the neophobic behavior displayed by animals presented with novel stimuli [39], with the assumption that more than 24-h exposure would increase the probability of fipronil bait being consumed. Hence, consumption would be greater on Day 2 exposure relative to Day 1. Fipronil bait was weighed to the nearest 0.1 g daily. Mice within the control groups (CDay1, CDay9, CDay15) received no fipronil bait and were presented with commercial rodent diet which was replenished *ad libitum*.

#### Post-exposure

At the conclusion of the exposure period, all remaining fipronil bait was removed and replaced with commercial rodent diet provided *ad libitum* for the remainder of the experiment. Mice were observed daily for health. During post-exposure, mice within test groups were exposed to larvae at one of three timepoints. This process is described explicitly below.

#### Tick attachment

Mice within the test groups, were assigned to one of three tick attachment timepoints: Day 1 (TDay1, CDay1); Day 9 (TDay9, CDay9); and Day 15 (TDay15, CDay15) post-exposure.

During tick attachment, larvae were applied to each mouse within a small feeding capsule made using ~¼ of a 1.5 ml plastic centrifuge tube. The capsules were used to (i) focus larval feeding in an isolated area to allow for direct observation; and (ii) improve larvae recovery by preventing mice from removing them through grooming. Capsules are a preferred method of containing and localizing ticks when facilitating blood-feeding [28–31]. Approximately 40 larvae were applied to each capsule to increase the probability of successful feeding and larvae recovery.

**Fig. 2:**
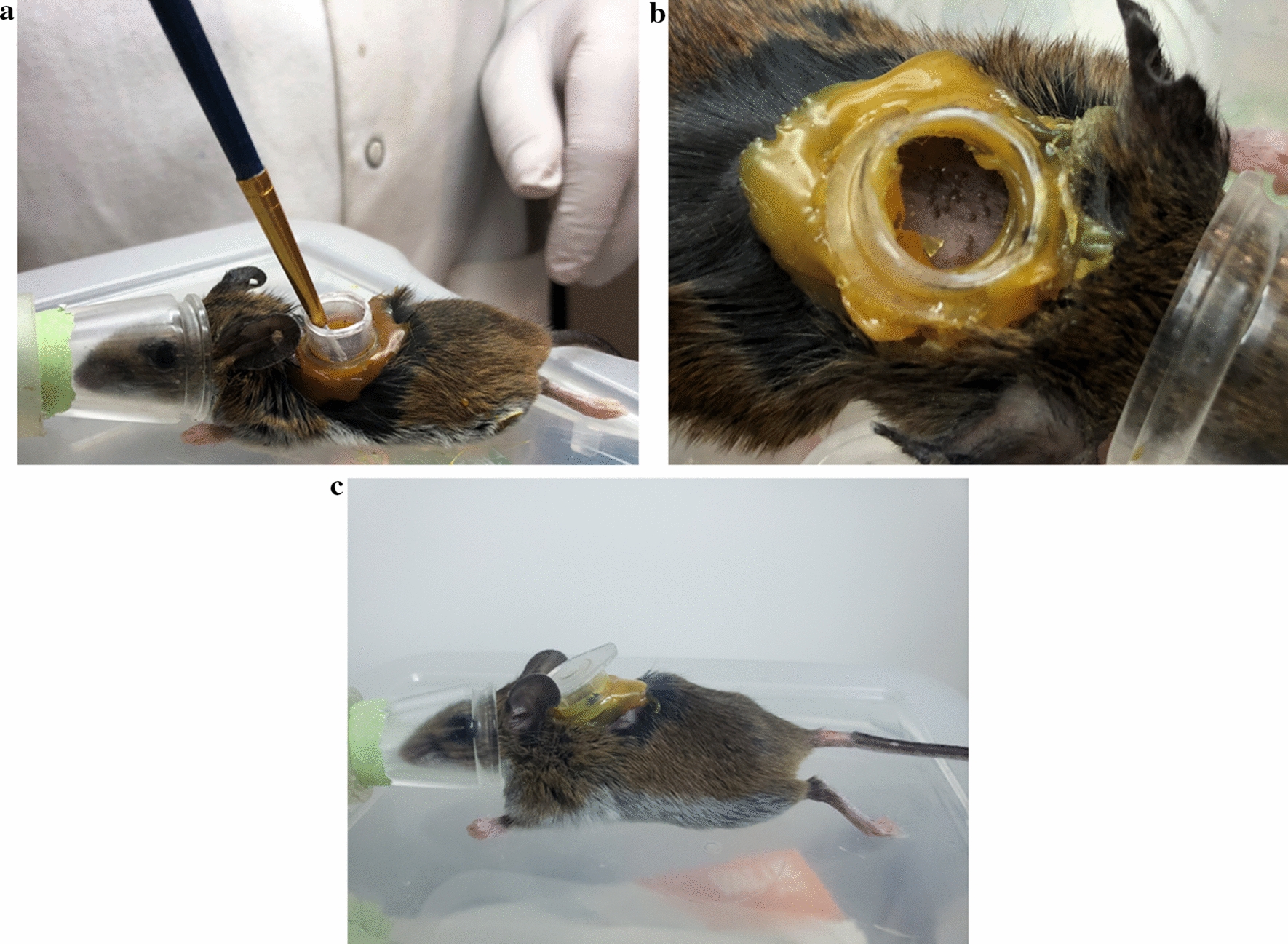
Mouse connected to the isoflurane nosecone. **a** Larvae being applied *via* fine-tipped paint brush. **b** Larvae within capsule. **c** Completed capsule with lid secured

Mice were individually placed into an induction chamber and anesthetized using an isoflurane vaporizer. The vaporizer was set to 4% isoflurane with an oxygen flow rate of 2 l/min. Once each mouse was anesthetized, it was removed from the induction chamber and attached to a nosecone (Fig. 2). The vaporizer and oxygen flow rate were then reduced to 1.5–2.0% and 0.5 l/min, respectively. Mice were monitored throughout the procedure to ensure that they were breathing normally.

A patch of fur between each anesthetized mouse’s shoulder blades was carefully trimmed to near the skin using an Equine FX^TM^ horse shearer (Conair Corp., Stamford, CT, USA). This was done to improve the ability to observe attached larvae during blood-feeding. The capsule was attached using a mixture of rosin and natural bees wax (3:1 ratio). The mixture was heated using a hot plate (IKA® C-Mag HS 7) until it was malleable enough to apply to the mice and was then allowed to cool for ~3 min. The mixture was applied to the rim of the capsule which was then secured to the shaved area of the mouse. This method is used routinely to blood-feed ticks in laboratory settings and did not significantly impact the behavior or longevity of ticks during this study.

While attaching larvae, the work surface was covered with a layer of white paper so that escaped larvae would be detectable. Double-sided tape was applied to the perimeter of each work surface, the base of each wall, and the perimeter of the doorway to prevent larvae from escaping the insectary. Tubes containing larval masses were placed into a white plastic tub lined with petroleum jelly to prevent escape. Active larvae displaying questing activity were selected from two larval masses. Forty larvae were transferred into each capsule using a fine-tipped paint brush (Fig. 2a, b). A plastic lid punctured with holes was secured to each capsule opening (Fig. 2c). The lids provided adequate air exchange while preventing larvae from escaping through the capsule opening. After completion of capsule and tick attachment, mice were removed from the nosecone and returned to their respective cages and monitored closely until fully recovered. A heat lamp (250W) was used to keep animals warm during recovery. Mice were monitored every 15 min for the remainder of the day following anesthesia.

#### Post-tick attachment

**Fig. 3:**
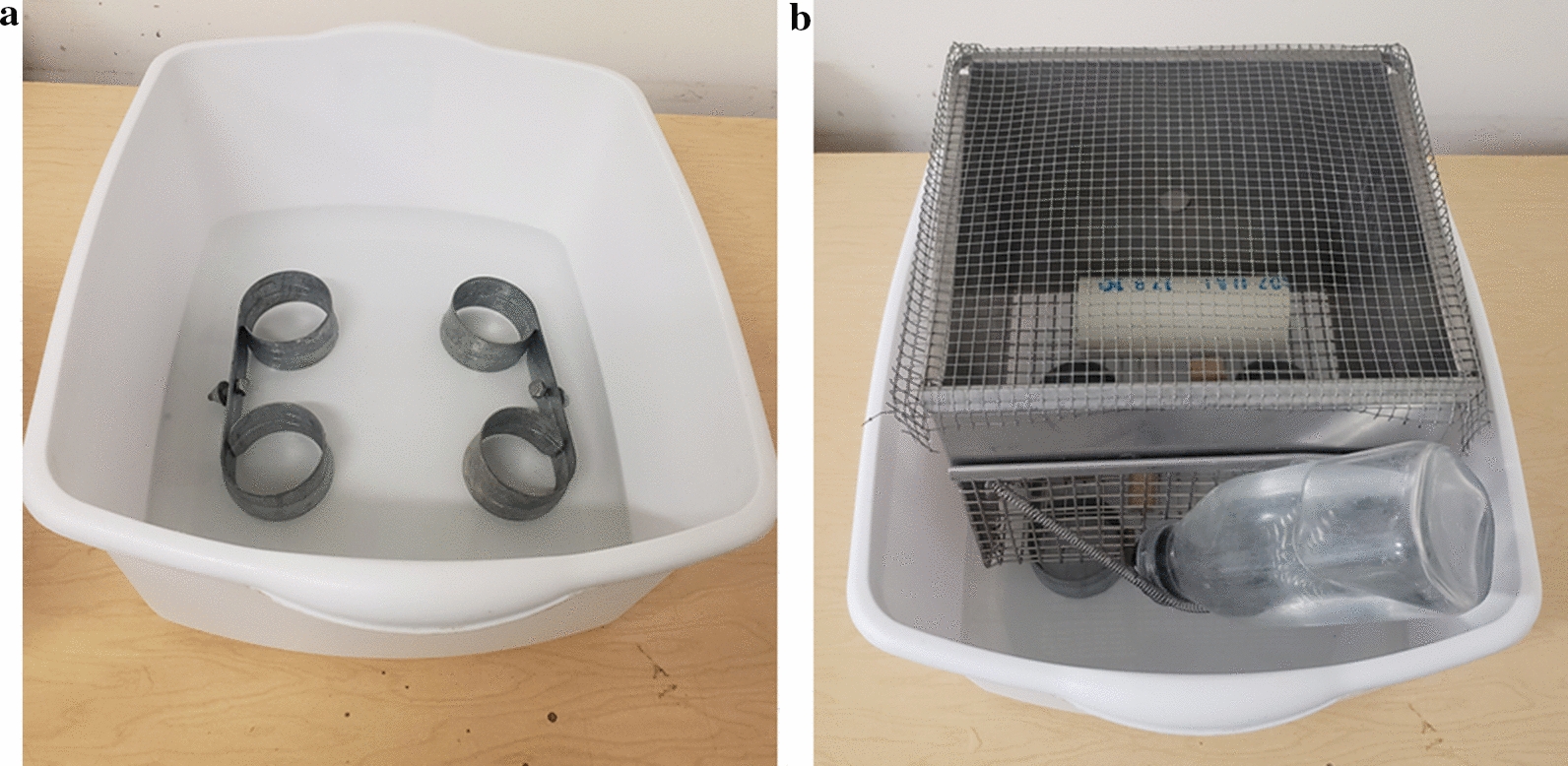
Cage and moat used during post-tick attachment. **a** moat with ~1.5 cm water and brackets used to suspend cage. The walls of the tubs were coated with petroleum jelly. **b** Cage, with food, water, and polyvinyl chloride (PVC) shelter, positioned within the moat

The bedding under each mouse cage was replaced with a plastic tub which was filled with ~1.5-cm of water (moat) (Fig. 3). The purpose of the moat was to collect replete larvae and prevent larvae from escaping. Metal brackets were positioned under each cage, serving as a base to keep the entire cage suspended above the water. Petroleum jelly was applied to the walls of the moat as an additional barrier to prevent larvae from escaping. At Day 2 post-tick attachment, mice were again anesthetized using the previously described method and the capsule lids were permanently removed in an effort to (i) observe larvae within the capsule during attachment and feeding, and (ii) allow larvae to exit the capsules after feeding to repletion and detaching.

The post-tick attachment period was commenced at Day 1 (TDay1, CDay1), Day 9 (TDay9, CD-9), and Day 15 (TDay15, CDay15) post-exposure. The day larvae were placed into capsules was defined as Day 0 post-tick attachment. After applying larvae to capsules on Day 0, larvae remained on the mice and were monitored continuously over the next 4 consecutive days (Day 1 to Day 4 post-tick attachment).

**Fig. 4:**
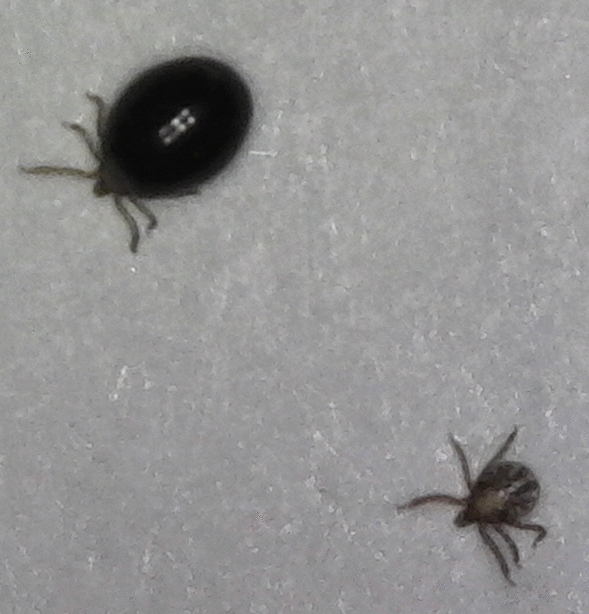
Comparison of unfed larvae (bottom) and replete larvae (top)
ii. At Day 2 (48-h) and Day 4 (96-h) post-tick attachment, each mouse was anesthetized using the previously described methods and placed individually under a microscope (2–4×) to observe the inside of the capsule. The inside of the capsule was carefully scanned for any observable, attached larvae. Attached larvae were those that were imbedded in the skin of the mice and attempting/having attempted to blood-feed. Attached larvae were examined closely and identified as non-engorged (flat or desiccated) or engorging (actively feeding and swollen). After identifying all observable larvae, representative images of the inside of the capsules were taken using a digital microscope (Plugable Technologies, Redmond, WA, US) connected to a portable laptop computer. The adhesive properties of the mixture were robust, with 59 of 60 capsules (98.3%) remaining on test mice throughout post-tick attachment. Capsules were carefully removed from mice at the conclusion of microscope observations on Day 4 post-tick attachment. To prevent pain and distress, capsules were slowly and gently removed while mice were anesthetized. Mice were then returned to holding.

During post-tick attachment, two methods of observing larvae were used: (i) collecting unfed or replete larvae from moats; and (ii) observing attached larvae within the capsules *via* microscopy. These methods were used to determine if fipronil bait prevented larvae from feeding to repletion and detaching from the host.

i. Immediately following tick attachment, each moat was scanned for larvae twice daily, and the status of each larva (unfed or replete) was recorded. After each scan was completed, and after all observable larvae were collected, the water within each moat was replaced. Replete larvae recovered during scans were engorged and black, resembling poppyseeds when observed with the naked eye [25], and typically floated on the surface of the water. A comparison of replete and unfed larvae can be seen in Fig. 4. Replete larvae were removed from the moats, washed thoroughly with distilled water, and allowed to dry on filter paper (7.0 cm). Unfed larvae removed from moats were placed into 90% ethanol and disposed of. Unfed larvae collected from moats were not included in efficacy estimates. Sanitized replete larvae were then placed into sample tubes and maintained in a desiccator under the same conditions previously described.

An explicit schedule detailing the dates of acclimation, exposure, post-exposure, tick attachment, and post-Tick Attachment for each test group is presented in Additional file 1.

#### Fipronil plasma concentration

Sixteen mice were selected for blood collection requiring euthanasia, to determine the fipronil concentration in plasma (CP). Mice were selected using a sequence generator (random.org). Mice were anesthetized in the same manner as described previously and euthanized using cervical dislocation in accordance with the United States Animal Welfare Act (AWA) recommended procedures [40]. Approximately 100 µl blood was collected from each animal *via* cardiac puncture using a 1cc syringe with 28-gauge needle. Blood samples were placed in a centrifuge and spun at 6100× *rpm* for 10 min.

Plasma was collected from mice selected from TDay9 (*n *= 4) and TDay15 (*n *= 4), at the conclusion of the respective post-tick attachment periods (Days 13 and 19 post-exposure). To obtain Day 1 CP, 4 extra-mice were exposed to fipronil bait for 48 h and plasma was collected at 24-h post-exposure. Hence, the 4 extra-mice were not utilized during the tick attachment or post-tick attachment period. Finally, 4 control mice were randomly selected for plasma collection. Plasma samples were stored at − 20 °C until analysis was conducted. Plasma samples were analyzed for CP of fipronil and fipronil metabolites (ng/ml) using a validated liquid chromatography-mass spectrometry (LC/MS) procedure with a limit of quantification (LOQ) of 1.25 ng/ml.

### Data analyses

#### White-footed mouse body weights

Body weights of all mice were recorded prior to fipronil bait exposure and at study termination. Differences in body weight between test groups (treatment *vs* control) and within each test group (initial weight *vs* final weight) were estimated using a Student’s t-test (*P* ≤ 0.05).

#### Fipronil bait consumption

Fipronil bait consumption was weighed daily for each mouse. We then estimated the total fipronil consumed by each mouse each day. The body weights taken prior to fipronil bait exposure were used to estimate total fipronil consumption in mg/kg for each mouse. Differences in fipronil consumption (mg/kg) between groups were estimated using a single-factor one-way analysis of variance (ANOVA; *P ≤* 0.05). Differences in daily fipronil consumption (mg/kg) recorded at Day 1 (24-h) and Day 2 (48-h) post-exposure, were estimated using a Student’s t-test (*P ≤* 0.05).

#### Tick observations

All larvae detaching from mice were collected from moats, and the total number and status of the larvae (unfed, replete) were recorded daily during the post-tick attachment period. Larvae within capsules were observed under the microscope and were counted and their status (non-engorged, engorging) determined at Day 2 and Day 4 post-tick attachment. Observed larvae were defined as: *Unfed*: flat larvae, showing no discernable blood meal, collected from moats; *Replete*: engorged, darkly colored larvae collected from moats; *Non-engorged*: attached larvae, which expired and desiccated and/or had no discernible blood meal, observed *via* microscopy; *Engorging*: attached, actively feeding, bloated larvae observed *via* microscopy.

#### Repletion efficacy

Efficacy of fipronil bait against larvae was evaluated in two ways: (i) whether it reduced the number of replete larvae collected in the moats; and (ii) whether it prevented larvae observable under the microscope from successfully detaching from the host. The rate of immature tick attachment is often low [30] and therefore the efficacy of fipronil bait in preventing larvae from feeding to repletion was calculated using the following equation described by Henderson & Tilton [41] to adjust efficacy to account for a loss of larvae within the control groups:

$$ Efficacy \left( \% \right) = 100 \times \left( {1 {-} \frac{Ta \times Cb}{Tb \times Ca}} \right) $$

where *T* is the treatment group, *C* is the control group, *b* is the mean initial number of larvae placed into capsule per mouse, and *a* is the mean number of replete larvae collected per mouse.

#### Attachment efficacy

The post-tick attachment microscope observations were used to estimate the number of larvae within capsules successfully detaching from mice. The total larvae successfully detaching from each mouse was estimated using the following formula:

Total successfully detached= Total attached (Day 2) – Total attached (Day 4)

The efficacy of the TS in preventing larvae from successfully detaching was calculated using the Henderson & Tilton equation [41], with the variables redefined as follows: *T*, treatment group; *C*, control group, *b*, total attached (Day 2); *a*, total successfully detached.

Differences in the number of (i) replete larvae collected from moats per test group; (ii) attached non-engorged and engorging larvae within capsules per test group; and (iii) larvae within capsules successfully detaching per test group, were analyzed using a Wilcoxon signed-rank test (*P *< 0.05). All statistical analyses were performed using JMP Statistical Software (Version 15) (Cary, NC, US).

### Good Laboratory Practice standards

This study was conducted according to EPA (1998) Good Laboratory Practice Standards (GLP) which are required for Federal Insecticide, Fungicide, and Rodenticide Act (FIFRA) pesticide registration (Title 40. Protection of Environment, Code of Federal Regulations Part 160 Good Laboratory Practice Standards). As per GLP requirements, an independent quality assurance unit monitored and inspected all components of the study.

## Results

### Body weights

The mean initial and final body weights for test groups are presented in Table 2. On average, each test group weight increased at the final weight relative to initial weight, with only females in CDay9 decreasing on average. Initial (Student’s t-test: *t*_(44)_= − 0.949, *P *= 0.347) and final (Student’s t-test: *t*_(44)_= 0.849, *P *= 0.400) body weights did not differ significantly when comparing treatment and control groups and no groups demonstrated significant weight loss over the course of the study. The final weight of treatment groups was significantly greater than the initial weight (Student’s t-test: *t*_(44)_= − 3.286, *P *= 0.002).

**Table 2:** Initial and final bodyweights (g) for white-footed mice within each test group (Mean ± SD)

Test Group	Sex	Initial weight (g)	Final weight (g)
CDay1	Male	20.2 ± 1.8	21.6 ± 2.6
	Female	17.9 ± 1.6	20.6 ± 1.6
CDay9	Male	19.9 ± 2.0	20.2 ± 2.2
	Female	18.2 ± 2.4	17.5 ± 3.2
CDay15	Male	18.6 ± 2.5	19.4 ± 2.3
	Female	19.6 ± 3.7	19.7 ± 3.2
TDay1	Male	19.1 ± 1.4	22.4 ± 2.7
	Female	17.2 ± 1.5	19.5 ± 1.1
TDay9	Male	18.7 ± 1.0	19.7 ± 1.4
	Female	17.3 ± 1.4	19.7 ± 2.4
TDay15	Male	20.2 ± 2.5	20.2 ± 2.0
	Female	18.2 ± 3.2	20.8 ± 1.8

### Fipronil bait consumption

Fipronil consumption was similar when (i) comparing Day 1 and Day 2 consumption, and (ii) comparing consumption between the three treatment groups. Fipronil consumption (mg/kg) did not differ significantly at Day 1 and Day 2 exposure (Student’s t-test: *t*_(44)_= 0.094, *P *= 0.926) and did not differ significantly between TDay1, TDay9, and TDay15 mice (ANOVA: *F*_(3, 27)_= 0.910, *P *= 0.408). Total fipronil bait consumption averaged between 6.0 g and 6.9 g for treatment group mice (Table 3).

**Table 3:** Fipronil bait consumption by treatment group mice (*n *= 30) (Mean ± SD)

Treatment group	Sex	Body Weight (g)	Consumption		
			Bait (g)	Fipronil (mg)	Fipronil (mg)/Body weight (kg)
TDay1	Male	19.1 ± 1.4	6.9 ± 2.1	0.35 ± 0.10	17.89 ± 3.97
	Female	17.2 ± 1.5	6.6 ± 1.5	0.33 ± 0.08	19.03 ± 2.82
	Overall	18.11 ± 1.7	6.8 ± 1.8	0.34 ± 0.09	18.46 ± 3.49
TDay9	Male	18.7 ± 1.0	6.5 ± 0.4	0.33 ± 0.02	17.47 ± 1.28
	Female	17.3 ± 1.4	6.1 ± 0.6	0.31 ± 0.03	17.85 ± 2.45
	Overall	18.0 ± 1.4	6.3 ± 0.6	0.32 ± 0.03	17.66 ± 1.96
TDay15	Male	20.2 ± 2.5	6.9 ± 1.3	0.35 ± 0.06	16.99 ± 1.22
	Female	17.3 ± 1.4	6.0 ± 1.0	0.30 ± 0.05	16.90 ± 4.12
	Overall	19.2 ± 3.1	6.5 ± 1.2	0.32 ± 0.06	16.94 ± 3.04

### Repletion of tick larvae

Fipronil bait had a significant impact on the number of replete larvae collected from moats at all timepoints post-exposure. Within all three treatment groups, fipronil bait resulted in efficacy of 100% with 0 replete larvae being collected from moats (Table 4). The numbers of replete larvae collected within moats in each treatment group, relative to each control group, were significantly different (Wilcoxon signed-rank test: *Z = *− 10.103, *P *< 0.0001). In the control groups (CDay1, CDay9, CDay15), a total of 502 replete larvae were collected from within the moats. Larvae did not feed to repletion until Day 3 post-tick attachment, with all replete larvae being collected at Day 3 and Day 4.

**Table 4:** Efficacy of fipronil bait in preventing larvae from feeding to repletion

Test group	Total no. of replete larvae		No of replete larvae recovered	Mean no. of replete larvae per animal ± SD	Repletion efficacy (%)
	Day 3 ^ab^	Day 4 ^ab^			
CDay1	77	55	132	13.2 ± 4.6	
CDay9	92	90	182	18.2 ± 4.0	
CDay15	95	93	188	18.8 ± 7.3	
Control total	264	238	502		
TDay1	0	0	0	0	100
TDay9	0	0	0	0	100
TDay15	0	0	0	0	100
Treatment total	0	0	0		100

^a^Days post-tick attachment

^b^No larvae fed to repletion prior to Day-3 post-tick attachment

### Attachment and detachment of tick larvae

**Fig. 5:**
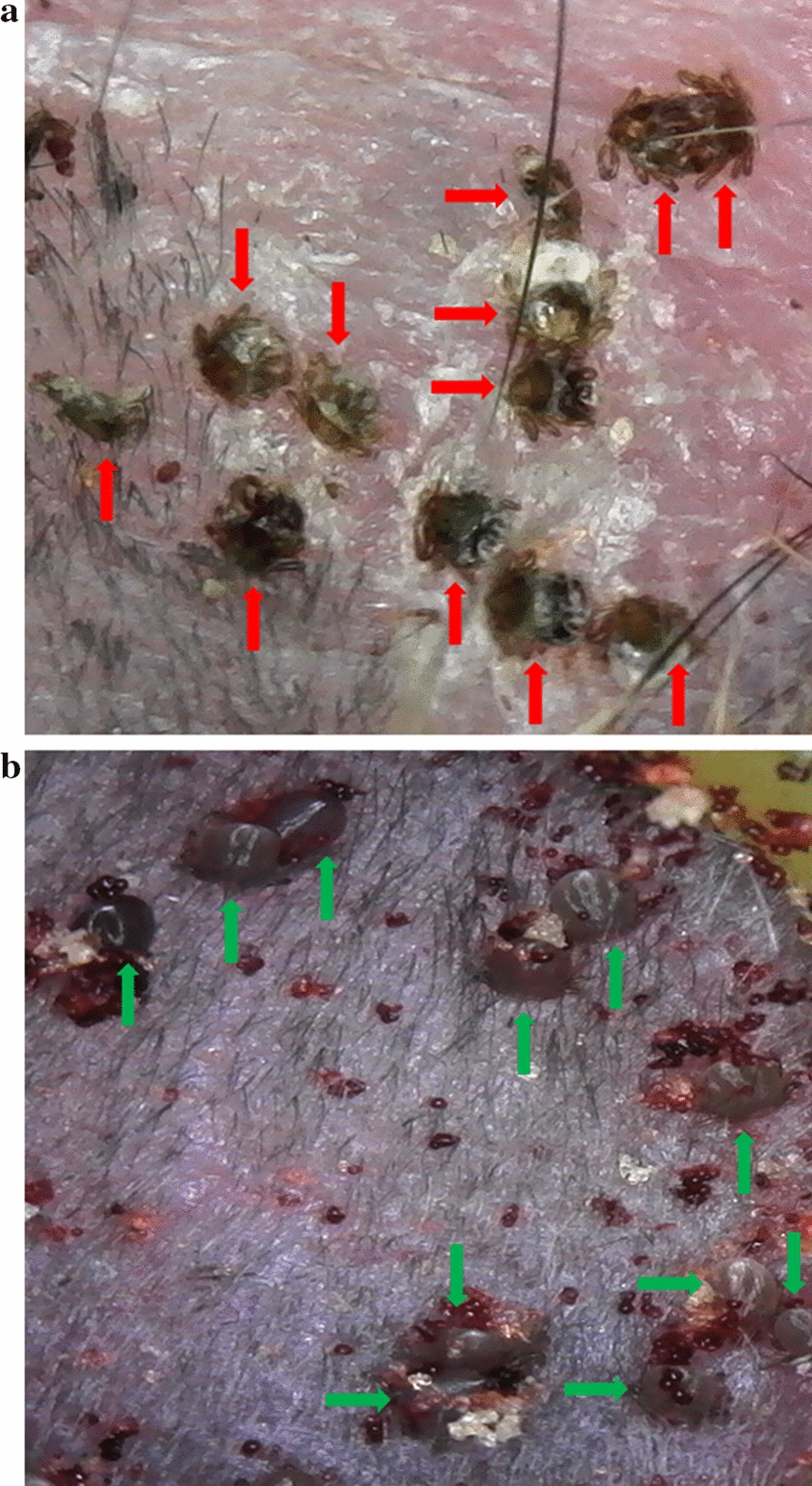
Larvae attached to a mouse exposed to fipronil bait (**a**) and a control mouse (**b**). These mice had larvae attached at Day-15 post-exposure. The photos were taken at Day-2 post-tick attachment. Red arrows indicate expired, desiccated larvae and green arrows indicate larvae that are engorging. The partial engorgement and presence of red feces indicate the actively feeding larvae. Photos were taken using a digital microscope (Plugable Technologies, Redmond, WA, USA)

Fipronil bait had a significant impact on the status of attached larvae within the capsules and the ability of these larvae to detach at all timepoints post-exposure. Within the treatment groups, at Day 2, a total of 368 larvae were observed attached within capsules of which 367 were non-engorged (99.7%) (Wilcoxon signed-rank test: *Z *= 7.067, *P *< 0.0001). At Day 4, within the treatment groups, all larvae observable within capsules (including the 1 Day 2 engorging larvae) were non-engorged, desiccated, and remained attached (Tables 5, 6). In the control groups (CDay1, CDay9, CDay15), a total of 368 larvae observable within the capsules were attached to mice on Day 2 post-tick attachment, of which 352 were engorging (95.7%) (Wilcoxon signed-rank test: *Z = *− 6.767, *P *< 0.0001). A total of 348 larvae (94.6%) successfully detached by the end of Day 4. The number of larvae failing to detach by Day 4 post-tick attachment was significantly greater within the treatment groups relative to control (Wilcoxon signed-rank test: *Z = *− 7.124, *P *< 0.0001). Fipronil bait demonstrated 100% efficacy in preventing larvae within capsules from successfully detaching from mice (Table 6). Figure 5 presents a Day 15 post-exposure comparison of non-engorged (expired) larvae on a treatment mouse and engorging (actively feeding) larvae on a control mouse (Day 2 post-tick attachment).

**Table 5:** Attached non-engorged and engorging larvae observable within the capsule under the microscope

Test group	Day 2 (post-tick attachment)		Day 4 (post-tick attachment)	
	Attached non-engorged	Attached engorging	Attached non-engorged	Attached engorging
CDay1	0.1 ± 0.3	9.0 ± 1.4	0	0.2 ± 0.4
CDay9	0.8 ± 0.9	12.7 ± 3.0	0.1 ± 0.3	0.2 ± 0.4
CDay15	0.7 ± 0.8	13.5 ± 3.6	0.2 ± 0.4	1.3 ± 1.5
TDay1	10.5 ± 2.8	0	10.8 ± 3.3	0
TDay9	13.2 ± 2.9	0	13.2 ± 2.9	0
TDay15	13.0 ± 4.0	0.1 ± 0.3	13.1 ± 4.0	0

*Note*: Number of larvae per mouse (arithmetic mean ± SD) at Day 2 and Day 4 post-tick attachment are indicated

**Table 6:** The efficacy of fipronil bait in preventing successful detachment of larvae (post-tick attachment)

Test group	No. of larvae attached (Day 2)	No. of larvae attached (Day 4)	No. of larvae successfully detached	Mean no. of successfully detached per mouse ± SD	Detachment efficacy (%)
CDay1	91	2	89	8.9 ± 1.4	
CDay9	135	3	132	13.2 ± 3.0	
CDay15	142	15	127	12.7 ± 2.6	
TDay1	105	108	0	0	100
TDay9	132	132	0	0	100
TDay15	131	131	0	0	100

### Fipronil plasma concentration

Fipronil sulfone, was the only fipronil metabolite detectable above the LOQ (1.25 ng/ml). CP averaged 948.9 ± 531.0, 101.2 ± 49.6, and 79.4 ± 15.6 ng/ml (arithmetic mean ± standard deviation (SD)) for mice euthanized on Day 1 (TD1), Day 13 (TD9) and Day 19 (TD15) post-exposure, respectively. No fipronil was detected in control group mice. A summary of the fipronil sulfone concentrations is presented in Table 7.

**Table 7:** The mean (± SD) consumption and CP for fipronil sulfone in 16 mice

Plasma collection day (post-exposure)	Fipronil consumption (mg/kg)	Fipronil concentration (ng/ml)
Control	0	0
Day 1	11.0 ± 3.5	948.9 ± 531.0
Day 13	16.8 ± 0.3	101.2 ± 49.6
Day 19	17.6 ± 4.6	79.4 ± 15.6

## Discussion

The results of this study suggest that, under laboratory conditions, a low dose fipronil bait (0.005%), presented orally to the primary pathogen reservoir (white-footed mice) for 48 hours, can potentially control 100% of blacklegged tick larvae blood-feeding at Day 1, Day 9 and Day 15 post-exposure. These results are an early indicator of the potential use of fipronil bait for *B. burgdorferi* (*s.s.*) prevention in the midwestern and northeastern USA. Under field conditions, successfully preventing larvae from feeding to repletion would reduce the influx of infected nymphs the following spring, reducing the risk of infected tick bites. Additional research may aid in determining key factors to maximize the success of this approach while also minimizing potential risks.

Future studies should be designed to answer additional questions regarding the efficacy of fipronil bait against larvae, which would aid in development of a field trial. First, the efficacy of fipronil bait should be evaluated at additional timepoints ranging from 21–42 days post-exposure. Although there were no rodenticides in the formulation used during this study, it was initially suggested that the bait be formulated with the first-generation anticoagulant warfarin at 0.025%, which is considered the nominal concentration presented in most commercially available rodenticides [42]. The theory was that many end-users would elect to remove the rodents, given the role that *Peromyscus* spp. play in Hantavirus [43] and Lyme disease [44] transmission. The debate about the use of an acaricide/rodenticide combination formulation was centered on the argument that mass die-offs of rodent species can result in the release of arthropod vectors into the surrounding environment, presenting an increased threat to humans [45, 46]. The post-exposure timepoints utilized during the present study span the relative days until death reported for first-generation anticoagulants, which average 5–7 days and up to 12 and 13 days for laboratory mice and rats, respectively [47]. One hundred percent (100%) of larvae were prevented from feeding to repletion even at Day 1, Day 9 and Day 15 post-exposure, indicating the potential use of fipronil bait as an acaricide-only product. Given that 100% efficacy was obtained during this study, we were unable to calculate the LD_50_ of low dose fipronil bait. Considering we observed a larva within TDay15 feeding at Day 2 post-tick attachment, it is likely that efficacy would decrease at least slightly within the coming weeks. The CP level recorded at Day 19 (79.4 ng/ml) indicates that fipronil sulfone might persist in mice over several weeks, and therefore efficacy at additional timepoints should be evaluated.

Future research should evaluate the efficacy of fipronil bait at variable exposure durations. As previously stated, mice were exposed to fipronil bait for 48 hours to compensate for potential neophobic reactions. However, our results indicated that neophobia may not be a significant issue as consumption did not differ significantly when comparing Day 1 and Day 2 consumption. This indicates that a mouse may be capable to consuming the necessary amount of fipronil within 24 hours to effectively control larvae, suggesting that this should be explored explicitly during future experiments. Additionally, prior research suggests that although white-footed mice may initially exhibit a neophobic response to new stimuli, they quickly become neophiliac [48]. This suggests that, under field conditions, a mouse may return to feed on fipronil bait repeatedly over > 2 consecutive days. If this were to occur, the CP in plasma of wild mice might be elevated enough for efficacy to be maintained for longer durations.

The results of the present study suggested that 0.005% fipronil bait, presented orally for 48-hours, posed minimal risk to mice. All mice remained healthy throughout the study, and no test groups exhibited weight loss. Average final weights were increased for all test groups and were significantly greater within the treatment groups. This was likely in part a byproduct of reduced stimulus, as these mice were normally group-housed in large enclosures while in holding. The significant increase in the weight of treatment mice could have additionally resulted from the removal of ectoparasites resulting from fipronil exposure. Although fipronil is considered moderately toxic when administered orally, as opposed to moderate to low toxicity when administered dermally/topically [49], the concentration of fipronil in the current bait is 19.4× and 9.7× lower than the bait utilized during prior work with fipronil [25]. The oral LD_50_ of fipronil for laboratory mice is ~97 mg/kg body weight. Given the concentration of fipronil in the bait (0.005%), a 20 g white-footed mouse would need to eat ~39 g of bait within 24 hours in order to reach the oral LD_50_, an improbable feat. However, additional studies, in which mice are exposed to fipronil bait chronically, could provide useful insights.

The results suggest that targeted white-footed mouse treatment with a fipronil-based oral acaricide could be economically advantageous. The desired end-use would be for pest control professionals and homeowners to be able to utilize the fipronil bait in a species-specific bait station. Ideally, a bait station could be placed in the field and remain for several weeks, without any need for maintenance such as replacement of topical wicks, as is required with the TCS bait box. Fipronil has been found to be a particularly effective compound when used for arthropod control. Researchers in Wyoming suggested that fipronil could be effectively applied at rates 100–200× less than alternative compounds when applied aerially for grasshopper control [50]. These researchers further hypothesized that the ability to apply fipronil at reduced concentrations may have mitigated toxicity to non-target species. Poche et al. [34] noted the above study and compared the application rates to that of a field trial in Kazakhstan utilizing a fipronil bait containing 0.005% fipronil. This study resulted in 100% efficacy against fleas (*Xenopsylla* spp.) infesting great gerbils (*Rhombomys opimus*) up to 80 days post-application with the bait being applied at a rate > 65× less than during the previously described study in Wyoming. Thus, specifically targeting the white-footed mouse with a fipronil bait presented in a species-specific bait station may significantly reduce the amount of labor required for the end-user and will exponentially reduce the amount of active ingredient being utilized relative to approaches such as blanket spraying. Future studies, including a large-scale field trial, could indicate the necessary application rates and provide more definitive insights into any potential economic or environmental advantages associated with this tick control approach.

Observing the larvae attached to mice *via* microscopy proved to be a useful addition to estimating the efficacy of fipronil bait. Larvae succumbing to fipronil remained attached to mice and therefore were not able to be collected from the water. Hence, this method allowed us to compare attached larvae within the treatment groups and control groups and confirm their condition. The results clearly demonstrate that larvae that attached to mice within the treatment groups were not able to feed to the extent that larvae attaching to mice within the control groups were and expired prior to reaching repletion and detaching. Although this method improved our ability to observe attached larvae, the number of replete larvae collected within moats was greater than the number observed under the microscope, indicating that a number of larvae attached to mice were not observable. Overall, this method proved useful in confirming the effectiveness of a systemic fipronil bait in controlling blood-feeding larvae. It should be noted that while the feeding capsules localized the larval feeding and prevented some larvae from escaping, a significant proportion (44.9%) of larvae were still never recovered. That is why the equation described by Henderson and Tilton [41] was utilized, allowing us to consider the loss of larvae within the control groups. Larvae were lost for several reasons such as escaping at the base of the capsules under mouse fur. While unfed larvae were recovered in the water moats, many specimens were never found. It was assumed that some larvae attached to feces or other debris in moats and were not found during scans. The difficulties associated with tick feeding have been described in previous studies [26, 30, 51]. Larvae are particularly challenging to recover because of their small size (≤ 0.8 mm in length). Future studies should consider creative modifications to further improve tick attachment and recovery under laboratory conditions.

As was the case during the fluralaner studies conducted by Pelletier et al. [26], in the present study the CP of fipronil for mice was highly variable, particularly at Day 1 post-exposure. The mean CP of fipronil was markedly lower at Day 1 post-exposure (948.9 ng/ml) when compared with the mean CP of fluralaner at Day 2 post exposure (0.005%= 13,815 ng/ml; 0.0.00125%= 4594 ng/ml); however, we should note that consumption by the Day-1 mice was low relative to the TDay9- and TDay15 mice. The CP of fluralaner dropped considerably at Day 28 (0.005%= 579 ng/ml; 0.00125%= 208 ng/ml) and Day 42 (0.005%= 46.7 ng/ml; 0.00125%= 52 ng/ml) post-exposure and these researchers observed no significant effect of treatment on feeding larvae at either of these timepoints [26]. In the present study, CP of fipronil sulfone dropped to 101.2 ng/ml (Day 13) and 79.4 ng/ml (Day 19). One hundred percent (100%) efficacy was obtained at Day 9 and Day 15 post-exposure, suggesting that fipronil bait effectively controlled larvae at CP< 101.2 ng/ml. The presence of fipronil sulfone in Day-19 plasma further suggests the need to determine efficacy and CP at additional timepoints post-exposure (Day 21 to Day 42) to estimate the lowest quantifiable CP at which significant larval efficacy can still be obtained.

The potential for fipronil bait to control blacklegged tick nymphs should also be investigated. Nymphs are primarily responsible for human Lyme disease transmission and will occasionally feed on small mammals, including white-footed mice [17–20]. Significantly reducing the number of nymphs could reduce the number of adult ticks capable of feeding on relevant reproductive hosts (i.e. *Odocoileus* spp. deer) and reduce the *B. burdorferi* transmission rate from nymphs to mice. However, considering the somewhat indiscriminate feeding behavior of nymphs, this would likely require integrated vector management, perhaps utilizing the current approach in conjunction with an oral acaricide to be administered to deer (*Odocoileus* spp.) [52]. Future researchers might consider investigating fipronil as a means of controlling nymphal and/or adult ticks feeding on deer. Additionally, researchers should consider evaluating the use of the current fipronil bait in controlling immature ticks attached to other potential mammalian hosts such as chipmunks. Controlling nymphs, larvae and adults simultaneously could potentially disrupt multiple blacklegged tick life stages, which would provide additional tick bite protection to humans and white-footed mice.

## Conclusions

Low dose fipronil bait, administered orally to white-footed mice for 48 hours, was efficacious in controlling blacklegged tick larvae attached to mice at Day 1, Day 9 and Day 15 post-exposure. Specifically, it was 100% efficacious in reducing replete larvae collected from moats and preventing larvae observed within capsules from detaching. This exceeds the efficacy requirement of 90% outlined by the EPA (OPPTS 810.3300) and suggests fipronil bait could eventually become a federally registered product to be utilized as a method of reducing tick abundance. To our knowledge, this is the first study to evaluate the efficacy of oral fipronil baits, presented to the primary *B. burgdorferi* (*s.s.*) reservoir (white-footed mice), against the blood-feeding primary *B. burgdorferi* (*s.s.*) vector (blacklegged tick). Explicit data regarding the level of efficacy and CP at various durations of exposure and timepoints post-exposure should continue to be collected and evaluated. These data would provide proper insights needed to develop a field trial or management plan. Given the efficacy that fipronil rodent baits have demonstrated against ticks, fleas and phlebotomine sand flies, a low dose fipronil bait, specifically targeting ticks infesting white-footed mice, could broadly target a variety of vector-host relationships, potential reducing transmission of a diverse group of zoonotic pathogens.

## Supplementary information

**Additional file 1: Table S1.** Study schedule providing the specific timing of acclimation, exposure, post-exposure, tick attachment, and post-tick attachment for each test group.

## Data Availability

Data supporting the conclusions of this article are included within the article and its additional files. The datasets generated during and/or analyzed during the present study are available from the corresponding author upon reasonable request.
